# Numerical Analysis of Airway Mucus Clearance Effectiveness Using Assisted Coughing Techniques

**DOI:** 10.1038/s41598-020-58922-7

**Published:** 2020-02-06

**Authors:** Shuai Ren, Wei Li, Lin Wang, Yan Shi, Maolin Cai, Liming Hao, Zihao Luo, Jinglong Niu, Weiqing Xu, Zujin Luo

**Affiliations:** 1 School of Automation Science and Electrical Engineering, Beihang University, Beijing, 100191 China; 2 Department of Orthopedics, Sixth Medical Center of PLA General Hospital, No. 6 Fucheng Road, Beijing, 100048 China; 3 North Automatic Control Technology Institute, Taiyuan, Shanxi 030006 China; 4 Department of Respiratory and Critical Care Medicine, Beijing Engineering Research Center of Respiratory and Critical Care Medicine, Beijing Institute of Respiratory Medicine, Beijing Chao-Yang Hospital, Capital Medical University, Beijing, 100043 China

**Keywords:** Cardiovascular biology, Interventional cardiology

## Abstract

Cough is a protective respiratory reflex used to clear respiratory airway mucus. For patients with cough weakness, such as chronic obstructive pulmonary disease, neuromuscular weakness disease and other respiratory diseases, assisted coughing techniques are essential to help them clear mucus. In this study, the Eulerian wall film model was applied to simulate the coughing clearance process through a computational fluid dynamics methodology. Airway generation 0 to generation 2 based on realistic geometry is considered in this study. To quantify cough effectiveness, cough efficiency was calculated. Moreover, simulations of four different coughing techniques applied for chronic obstructive pulmonary disease and neuromuscular weakness disease were conducted. The influences of mucus film thickness and mucus viscosity on cough efficiency were analyzed. From the simulation results, we found that with increasing mucus film thickness and decreasing mucus viscosity, cough efficiency improved accordingly. Assisted coughing technologies have little influence on the mucus clearance of chronic obstructive pulmonary disease models. Finally, it was observed that the cough efficiency of the mechanical insufflation-exsufflation technique (MIE) is more than 40 times the value of an unassisted coughing technique, which indicates that the MIE technology has a great effect on airway mucus clearance for neuromuscular weakness disease models.

## Introduction

As a protective respiratory reflex, cough clears mucus from the respiratory tract and keeps it clean and unobstructed. A normal cough has four phases^1–3^. First, the airways are irritated by foreign matter; then, the inspiration phase is conducted by the diaphragm and larynx muscles contracting, which is followed by the compression phase that combines closing the glottis and contracting abdominal and thoracic muscles; finally, the expulsion phase is performed through reopening the glottis and contracting expiratory muscles suddenly and forcefully, respectively.

**Figure 1:**
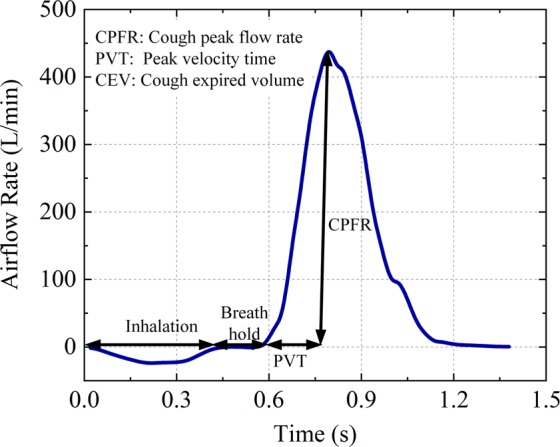
Cough airflow rate. The cough process begins with a short inhalation that follows a breath-holding process before opening the glottis. Then, the airflow reverses direction and rapidly increases until the maximum value is reached. Finally, the airflow gradually declines toward zero.

The coughing process can be characterized by three key parameters: cough expired volume (CEV), cough peak flow rate (CPFR) and peak velocity time (PVT)^4–8^. These parameters provide good insight into the flow behavior in the coughing process. Figure 1 shows that there is a short inhalation process before expulsion, and then a high acceleration of the flow rate appears, followed by a decay. The total duration of coughing is approximately 0.4–0.5 s^5^.

Bronchial mucus, which is produced by mucous cells, goblet cells and Clara cells in bronchial trees, presents a heterogeneous and pseudoplastic fluid property whose viscosity may be greatly reduced by increasing the shear stress^9–12^. Healthy mucus thickness is approximately 30 µm and can be easily transportable through ciliary swing because of its low viscosity and elasticity^9,13^. However, infections and bronchitis can increase mucus thickness and viscosity, resulting in mucus transport difficulty^14,15^.

During a normal cough, the high airflow velocity creates a high shear stress that shears secretions and foreign matter off the bronchial wall and propels them toward the larger airways and trachea. A cough is considered effective if CPFR is >160 L/min^16,17^. However, because of airflow obstruction, patients with chronic obstructive pulmonary disease (COPD) may present with weak cough and airway mucus deposition. Neuromuscular disease (NMD) and emphysema may cause cough ineffectiveness because of respiratory muscle weakness^18,19^.

The current assisted coughing techniques recommended for airway clearance mainly include manually assisted coughing (MA)^20^; mechanical insufflation (MI), which produces a cough after the inspiration supplied by a ventilator^21^; mechanical exsufflation (ME), which presents the negative pressure at the end of inspiration^22^; and mechanical insufflation/exsufflation (MIE), which promotes maximal lung inflation by positive pressure followed by a sudden switch to negative pressure to create a high airflow^23^.

Most previous studies have concentrated on mucus clearance and droplet dispersion through experimental methods^24–26^. Along with the accuracy and applicability of respiratory airflow simulation that have been verified through experiments, some computational fluid dynamics (CFD) simulation studies have successfully been applied in calculating airway wall shear stress and modeling coughing clearance processes in upper airways and droplet dispersion^27–31^. In a recent study, the Eulerian wall film (EWF) model was successfully applied in the mucus clearance process in the trachea and airway model^32,33^. However, CFD studies that simulate the bronchial mucus clearance process with assisted coughing techniques have not been found in the literature. Therefore, in this study, the EWF model was used to simulate the coughing clearance process for four different coughing techniques. Additionally, the influences of mucus viscosity and thickness on cough efficiency (CE) for NMD and COPD patients were studied in this paper.

## Methods

### Eulerian wall film model

Considering the small temperature change during the cough process in respiratory airways, only the continuity Eq. (1) and momentum Eq. (2) are solved, which are presented as follows:

1 $${\rm{div}}\overrightarrow{v}=0$$

2 $$\frac{d\overrightarrow{v}}{dt}=\overrightarrow{g}-\,\frac{1}{\rho }{\rm{div}}p+\upsilon {\nabla }^{2}\overrightarrow{v}$$

where $\overrightarrow{v}$ and $\overrightarrow{g}$ represent the velocity and gravity vectors, respectively, *p* is the air pressure, and *ρ* and *υ* are the air density and kinematic viscosity, respectively,

The mucus film continuity equation is as follows:

3 $$\frac{\partial h}{\partial t}+{\nabla }_{s}\cdot [h{\overrightarrow{v}}_{l}]=\frac{{\dot{m}}_{s}}{{\rho }_{l}}$$

where *h* is the film height, ${\overrightarrow{v}}_{l}$ and *ρ*_*l*_ represent the mean mucus film velocity and film density, respectively, *ṁ*_*s*_ represents the mass source per unit of wall surface, and ∇_S_ represents the surface gradient operator.

The mucus film momentum equation is as follows:

4 $$\frac{\partial h{\overrightarrow{v}}_{l}}{\partial t}+{\nabla }_{s}\cdot (h{\overrightarrow{v}}_{l}{\overrightarrow{v}}_{l})=-\,\frac{h{\nabla }_{s}[p-\rho h(\overrightarrow{n}\cdot \overrightarrow{g})-\sigma {\nabla }_{s}\cdot ({\nabla }_{s}h)]}{{\rho }_{l}}+{\overrightarrow{g}}_{\tau }h+\frac{3}{2{\rho }_{l}}{\overrightarrow{\tau }}_{la}-\frac{3\nu }{h}{\overrightarrow{v}}_{l}+\frac{\dot{q}}{{\rho }_{l}}$$

where $\overrightarrow{n}$ represents the normal vector, *σ* is the surface tension, ${\overrightarrow{g}}_{\tau }$ represents the gravity component parallel to the mucus film, ${\tau }_{la}$ represents the viscous shear force at the air and mucus film interface, *ν* is the kinematic viscosity, and $\dot{q}$ is the momentum source term associated with droplet interactions.

### Non-newtonian viscosity model of mucus layer

Because of the existence of macromolecule mucin polymers, mucus presents non-Newtonian fluid properties, which is called the shear-thinning phenomenon. Based on previous experimental studies, the mucus viscosity *μ* is a function of the shear rate *γ*, which is shown in Eq. (5)^13,34,35^.

5 $$\mu =\{\begin{array}{ll}{\mu }_{min} & \gamma  > {\gamma }_{max}\\ a\cdot {\gamma }^{b} & {\gamma }_{min} < \gamma  < {\gamma }_{max}\\ {\mu }_{max} & \gamma  < {\gamma }_{min}\end{array}$$

where *μ*_*min*_ and *μ*_*max*_ represent the minimum and maximum viscosity values, respectively. Similarly, *γ*_*min*_ and *γ*_*max*_ represent the minimum and maximum shear rates, respectively. In addition, *a* and *b* are constant values that are 2.52 and −0.85, respectively.

### Turbulence model

During the cough process, the Reynolds number (*Re*) generated by the flow in a narrow human airway may be as high as 10,000, resulting in turbulence. According to previous studies, the *k–ω* model combined with the shear stress transport (SST) submodel is recommended for airflow simulation in human airways^27,36^. Therefore, the *k–ω* model and SST submodel are used in this study.

### Boundary conditions

**Figure 2:**
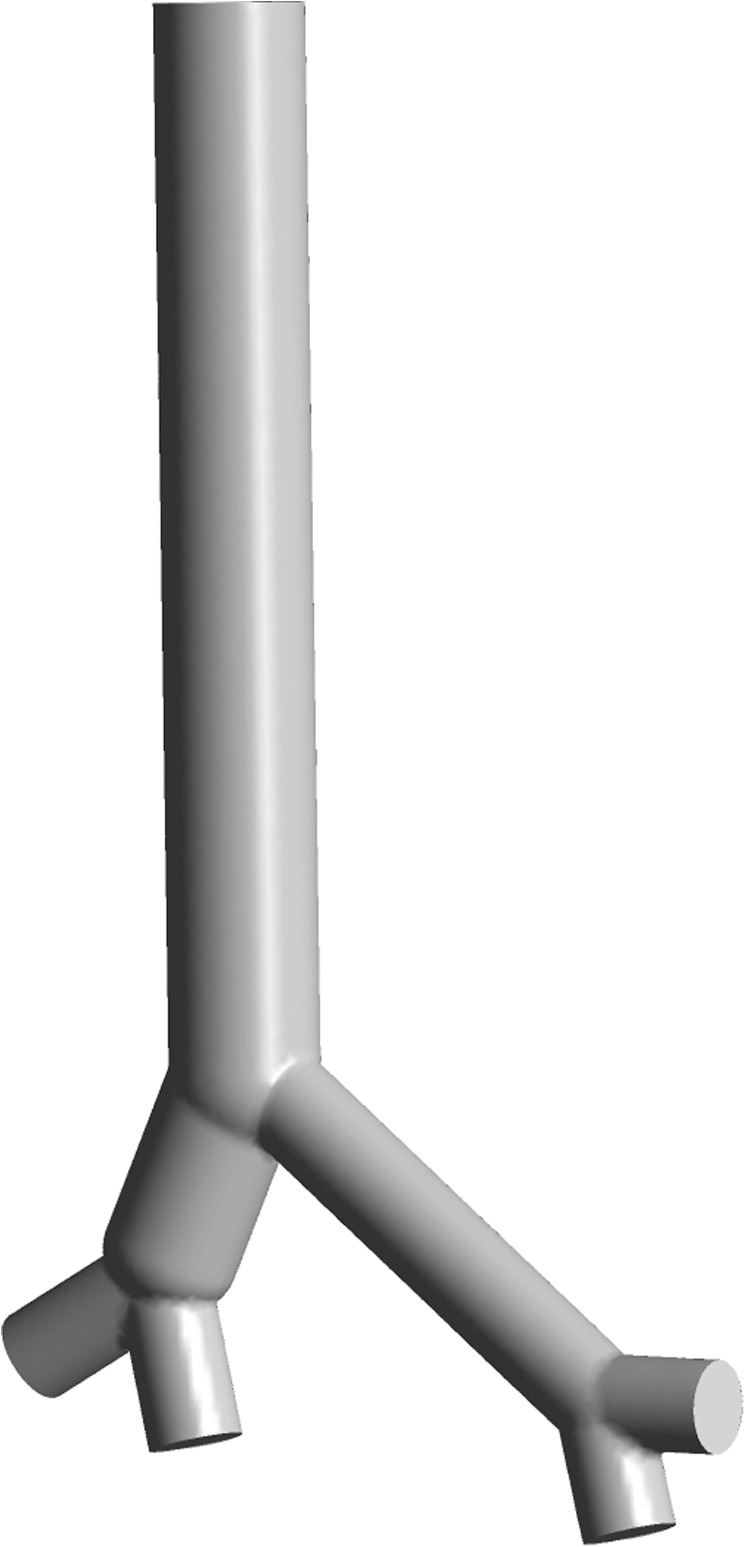
The 3D geometry used in this study. There are four inlets and one outlet in the geometry. The end faces of the bronchus and trachea are set as the inlets and outlet, respectively.

The airflow is considered an incompressible ideal gas whose density and viscosity are 1.139 *kg/m*^3^ and 1.89 × 10^5^ *Pa·s* (at 37 °*C* and 100 *kPa*), respectively. Airway structure parameters obtained from ref. ^37^ are used to generate the 3D geometry by using Gambit software. Because mucus transport by airflow and mucus deposition are higher in larger airways than in peripheral airways and the airway generations from 3 to 23 are far less than 1 *cm*, only the airway generations from 0 to 2 are considered in this study. The 3D geometry is shown in Fig. 2. Structured and unstructured meshes are combined to improve the accuracy. The number of cells was 164845. The air flows from the terminal of airway generation 2 to the top of airway generation 0. The inlet and outlet boundary conditions are set as the mass flow inlet and pressure outlet, respectively. The wall was considered stationary and no slip in all situations. Gravity was considered as a vertical state.

The mass flow rate is parameterized and coded by Fluent UDF with data taken from refs. ^5,19,22,38^. The cough airflow rate is generated through Gupta’s model^5^, which is based on gamma probability distribution functions and is presented below:

6 $$\begin{array}{ccc}\bar{M} & = & \frac{Flowrate}{CPFR};\,\tau =\frac{Time}{PVT};\\ \bar{M} & = & \frac{{{a}_{1}}^{\ast }{\tau }^{{{b}_{1}}^{\ast }-1}{e}^{(\frac{-\tau }{{{c}_{1}}^{\ast }})}}{\varGamma ({{b}_{1}}^{\ast }){{c}_{1}}^{\ast {{b}_{1}}^{\ast }}}\,{\rm{f}}{\rm{o}}{\rm{r}}\,\tau  < 1.2;\\ \bar{M} & = & \frac{{{a}_{1}}^{\ast }{\tau }^{{{b}_{1}}^{\ast }-1}{e}^{(\frac{-\tau }{{{c}_{1}}^{\ast }})}}{\varGamma ({{b}_{1}}^{\ast }){{c}_{1}}^{\ast {{b}_{1}}^{\ast }}}+\frac{{{a}_{2}}^{\ast }{(\tau -1.2)}^{{{b}_{2}}^{\ast }-1}e(\frac{-(\tau -1.2)}{{{c}_{2}}^{\ast }})}{\varGamma ({{b}_{2}}^{\ast }){{c}_{2}}^{\ast {{b}_{2}}^{\ast }}}\,{\rm{f}}{\rm{o}}{\rm{r}}\,\tau \ge 1.2\\ {{a}_{1}}^{\ast } & = & 1.680;\,{{b}_{1}}^{\ast }=3.338;\,{{c}_{1}}^{\ast }=0.428;\\ {{a}_{2}}^{\ast } & = & \frac{CEV}{PVT\times CPFR}-{{a}_{1}}^{\ast };\,{{b}_{2}}^{\ast }=\frac{-2.158\times CEV}{PVT\times CPFR}+10.457;\\ {{c}_{2}}^{\ast } & = & \frac{1.8}{{{b}_{2}}^{\ast }-1};\\ CEV(L) & = & 0.0204CPFR(L/s)-0.043\,{\rm{f}}{\rm{o}}{\rm{r}}\,{\rm{f}}{\rm{e}}{\rm{m}}{\rm{a}}{\rm{l}}{\rm{e}},\,CEV(L)\\  & = & 0.138CPFR(L/s)+0.2983\,{\rm{f}}{\rm{o}}{\rm{r}}\,{\rm{m}}{\rm{a}}{\rm{l}}{\rm{e}};\\ PVT(ms) & = & 3.152CPFR(L/s)+64.631\,{\rm{f}}{\rm{o}}{\rm{r}}\,{\rm{f}}{\rm{e}}{\rm{m}}{\rm{a}}{\rm{l}}{\rm{e}},\,PVT(ms)\\  & = & 1.360{\rm{C}}{\rm{P}}{\rm{F}}{\rm{R}}(L/s)+65.860\,{\rm{f}}{\rm{o}}{\rm{r}}\,{\rm{m}}{\rm{a}}{\rm{l}}{\rm{e}}.\end{array}$$

### Numerical solution

A pressure-based solver with a semi-implicit method is used to solve the pressure–velocity coupling scheme^39^. Because of the high precision in calculating the hexahedral meshes, the quadratic upwind interpolation scheme is adopted to solve the turbulence and momentum equations^40^. The EWF model discretization equations are solved through a second-order upwind scheme. The numerical simulations are conducted using ANSYS 17.0.

### Cough efficiency

The “cough efficiency (*CE*)” index, which is shown in (7) from ref. ^32^, is used to evaluate the mucus clearance effect through cough.

7 $$CE=\frac{{m}_{R}}{{m}_{T}}\times 100 \% $$

where *m*_*R*_ represents the removed mucus mass through a single cough and *m*_*T*_ represents the total mucus mass before the cough. The CE simulation results of healthy, NMD and COPD models are compared using the cough airflow parameters from refs. ^19,22,36^. The influences of different kinds of coughing techniques, mucus layer thickness and the viscosity reduction effect are also examined.

## Results

### Model validation

The airway model used in this paper was validated to be feasible by the published numerical results of Green^29^. The boundary conditions in the CFD simulations are equivalent to those used in Green’s paper.

**Figure 3:**
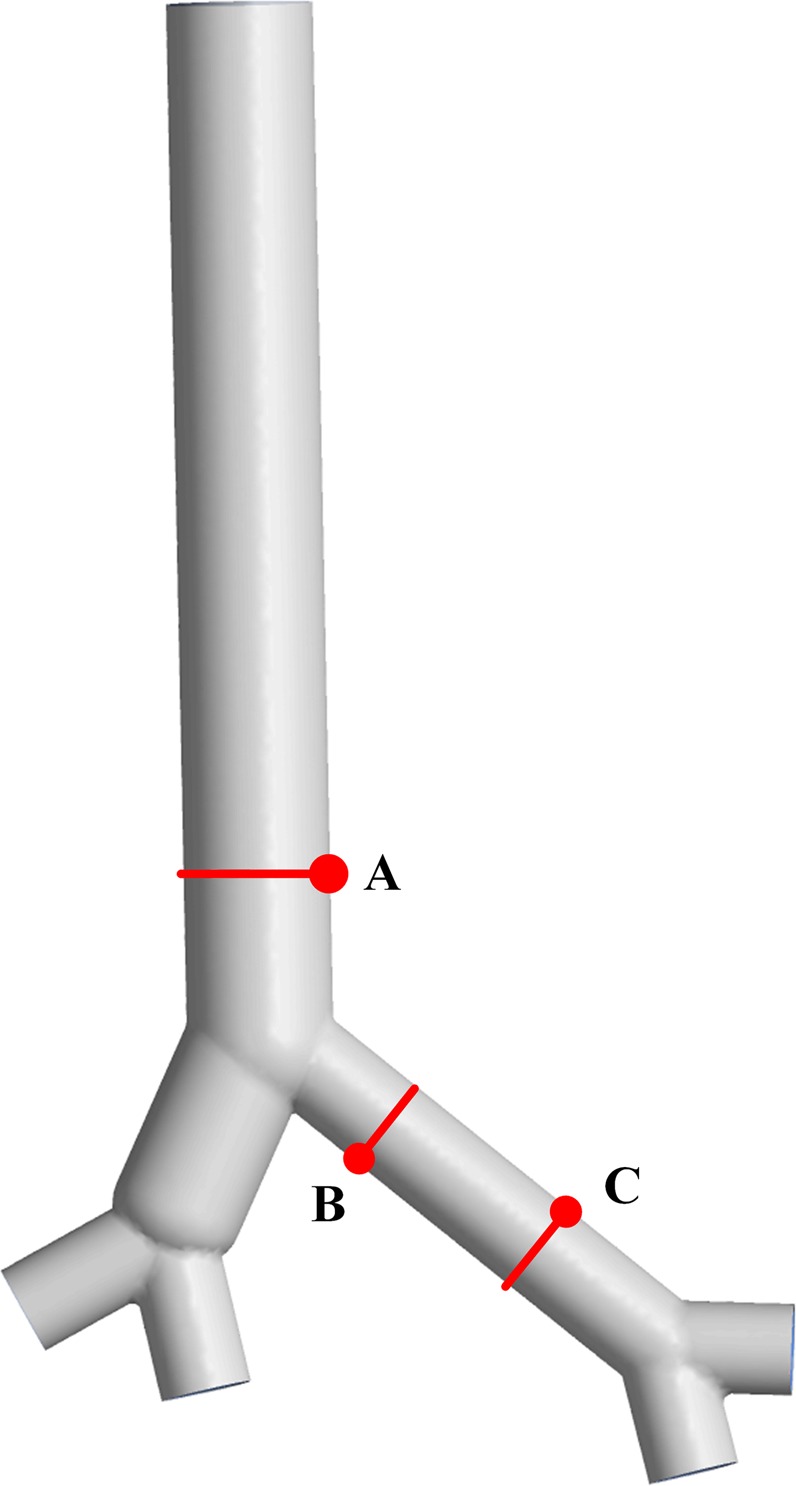
The geometry model and measuring locations. Location (**A**) is in the trachea, and locations (**B,C**) are in the left bronchus.

**Figure 4:**
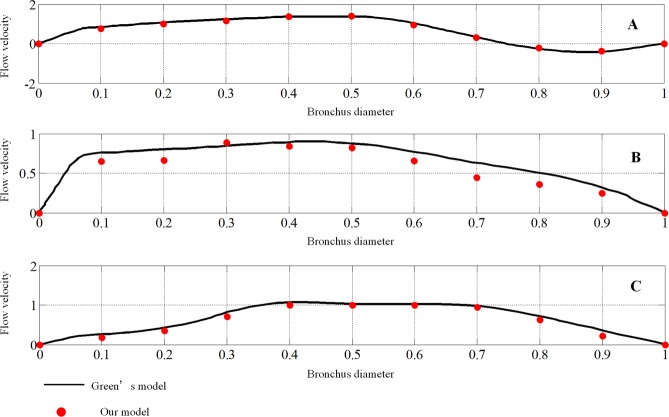
Comparison of mean velocity for locations (**A–C**). The velocities of eleven points are selected from the nondimensional local bronchus diameter at intervals of 0.1 for each location. The black lines represent the nondimensional flow velocity in Green’s model, and the red dots represent our results.

The velocities of the three locations shown in Fig. 3, just like those adopted in Green’s model, are calculated. The velocities are made nondimensional with the outflow bulk velocity. The locations are made nondimensional with the local bronchus diameter. A comparison of the results for an expiratory flow rate of 1 *L/s* under steady conditions is shown in Fig. 4.

### Influence of assisted coughing techniques

**Figure 5:**
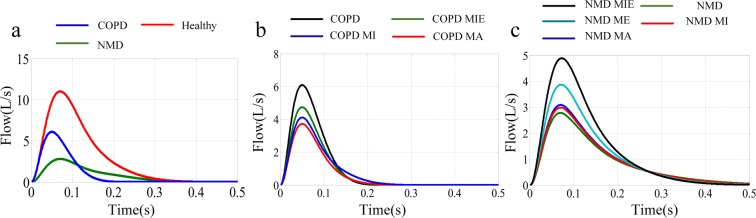
(**a**) Airflow rate for healthy, COPD and NMD models. The cough peak flow rate of the healthy model is obviously higher than that of the COPD and NMD models. There is a much more rapid decline in flow in the COPD model. (**b**) Airflow rate for COPD patients with MA, MI and MIE coughing techniques. (**c**) Airflow rate for NMD patients with MA, MI, ME and MIE coughing techniques.

**Figure 6:**
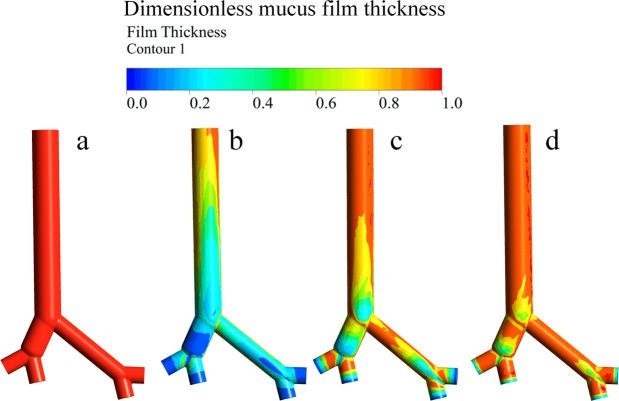
Dimensionless mucus film thickness without assisted coughing techniques: (**a**) before the cough process, (**b**) after the cough process for the healthy model, (**c**) after the cough process for the COPD model, (**d**) after the cough process for the NMD model.

The CE values of the four coughing techniques (MA, MI, ME, MIE) mentioned in the introduction section are compared among healthy, NMD and COPD models. The initial mucus film thickness was set to 30 *µm*. The relationships between cough flow with and without assisted coughing techniques and time are shown in Fig. 5. The dimensionless mucus film thickness during the cough process without assisted coughing techniques for healthy, COPD, and NMD models is shown in Fig. 6. Table 1 presents the CE values of healthy, NMD and COPD models with and without assisted coughing techniques.

**Table 1:** Cough efficiency (CE).

CE (%)	UA	MA	MI	ME	MIE
Healthy	56	—	—	—	—
COPD	12	7.54	8.45	—	9.72
NMD	0.16	0.59	0.64	2.65	9.82

Figure 5 shows that the coughing airflows of COPD and NMD models are greatly reduced compared with that of the healthy model. Figure 6 shows that there are great differences among healthy, COPD and NMD models without assisted coughing techniques.

### Influence of mucus film thickness

**Figure 7:**
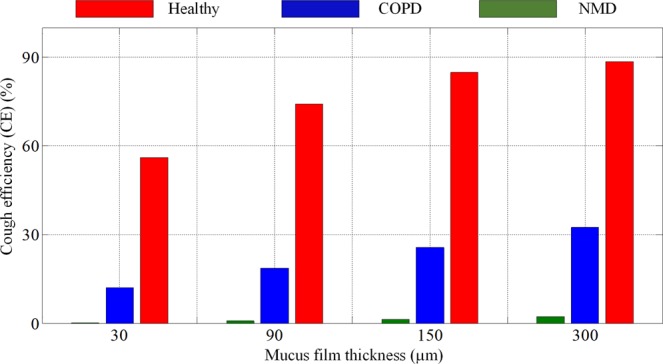
The CE values of healthy, COPD and NMD models for different mucus film thicknesses. The CE values of healthy, COPD and NMD models are marked red, blue and green, respectively. The cough efficiencies increase with increasing mucus film thickness for the healthy, COPD and NMD models.

In endobronchial disease situations, the mucus film thickness may be more than 10 times greater than healthy values^17^. For COPD, chronic bronchitis may be associated with significant mucus production, while emphysema may be associated with minimal mucus production. Therefore, the simulated mucus film thickness is changed from 30 to 300 *µm* in this study. Comparisons of the CE values of healthy, COPD and NMD models under different mucus film thicknesses are presented in Fig. 7. For the healthy model, the CE values increase with increasing mucus film thickness. The growth trend slows gradually with increasing thickness. However, the CE values improve to a small extent with increasing mucus film thickness for the COPD and NMD models.

**Figure 8:**
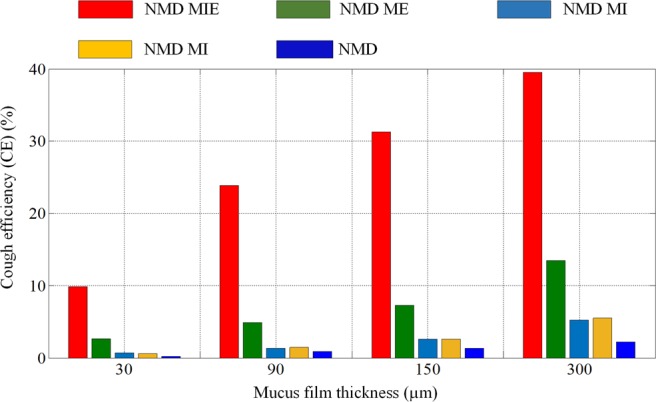
The CE values of NMD models for different mucus film thicknesses. The CE values of NMD patients with MA, MI, ME and MIE coughing techniques are marked brown, cyan, green and red, respectively. With increasing mucus film thickness, the cough efficiencies of all models demonstrate some improvements. In particular, the cough efficiency of the MIE technique is more than 40 times the value of an unassisted cough.

The CE values of NMD models with different assisted coughing techniques and different mucus film thicknesses are compared and shown in Fig. 8. Compared with the other three techniques, the MIE technique has an obvious effect on the CE values of NMD models. In particular, when the mucus film thickness reaches 300 µm for some diseases, the CE of MIE is more than 40 times the value of unassisted coughing. Additionally, the ME technique has little promotion effect on CE values.

### Influence of mucus viscosity

**Figure 9:**
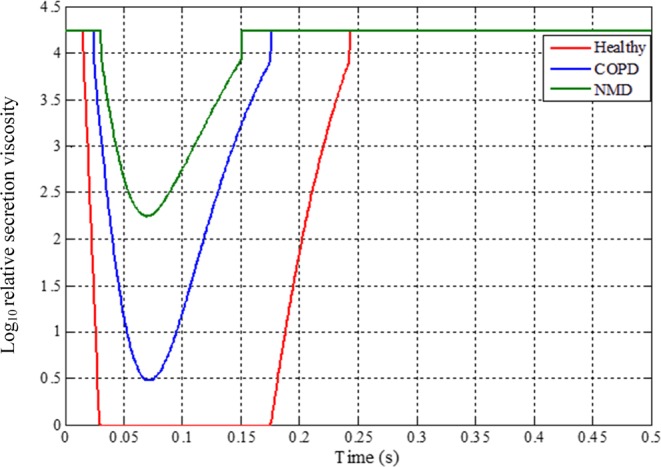
Variation of viscosity with time for healthy, COPD and NMD models during the cough process (relative mucus viscosity = mucus viscosity/water viscosity (0.001 *pa·s*, 20 °*C*)). The curves for healthy, COPD and NMD models are marked red, blue and green, respectively. For the healthy model, the viscosity decreases to the same level as that of water because of the high airflow produced in cough behavior.

The effect of a reduction in mucus viscosity on CE was analyzed in this study. The results shown in Fig. 9 present the variation of viscosity with time in a single cough process for healthy, COPD and NMD models. The viscosity of water is used for comparison. A logarithmic operation is applied to visualize the results.

**Figure 10:**
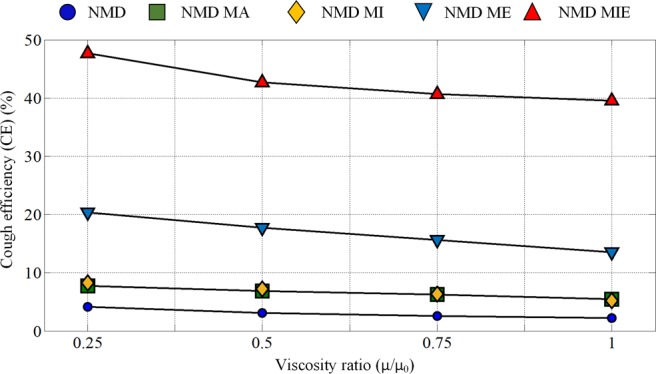
The CE value variations with mucus viscosity reduction (*μ*: current mucus viscosity; *μ*_0_: initial mucus viscosity; mucus film thickness: 300 *um*). Four viscosity ratio values (0.25, 0.5, 0.75 and 1) are investigated.

The CE values of NMD models with different assisted coughing techniques and different mucus viscosities are compared and shown in Fig. 10. The ratio of current mucus viscosity to initial mucus viscosity is used to represent the viscosity variation. Four ratio values (0.25, 0.5, 0.75, 1) are adopted in this study.

## Discussion

This paper conducted simulations of mucus clearance, which is a gas-liquid two-phase flow phenomenon, under different assisted cough techniques using a computational fluid dynamics (CFD) method. Assisted coughing techniques such as MA, MI and MIE have little influence on the coughing airflows of COPD models. However, for NMD models, the assisted coughing technologies could obviously improve the peak cough flow rate. Healthy models could clear mucus effectively through cough. However, for COPD and NMD models without assisted coughing techniques, a large amount of mucus was still attached to the airway wall surface.

Table 1 shows that the cough efficiency (CE) of the healthy models is approximately 56%, while that of the COPD and NMD models is only 12% and 0.16%, respectively. This indicates that COPD and NMD diseases could tremendously reduce CE. With the help of assisted coughing technologies, the CE of NMD models improves obviously; additionally, the CE is more than one hundred and fifty times the original value when using MIE. This indicates that MIE could greatly benefit mucus clearance for NMD patients. However, there is no obvious change in the CE values of COPD models when using assisted coughing techniques, which is consistent with previous results^19^.

The CE values increase with increasing mucus film thickness caused by wall shear stress under the same cough parameters and mucus viscosity, which is consistent with empirical knowledge. The results shown in Figs. 7 and 8 indicated that the MIE technique has a great effect on airway mucus clearance with increasing mucus film thickness caused by some diseases.

The variation in mucus viscosity shown in Fig. 9 is related to the different shear rates caused by the airflow. The shear-thinning phenomenon is obvious in healthy models compared with COPD and NMD models. The viscoelastic properties of mucus have a great influence on mucus clearance. Only the shear-thinning property of mucus has been considered in the current mucus layer model.

Figure 10 shows that reducing the mucus viscosity could improve the CE values to some extent. For NMD subjects, manually assisted (NMD MA) and mechanical insufflation (NMD MI)-assisted coughing techniques have little influence on mucus clearance, even though the mucus viscosity is reduced. However, for the mechanical exsufflation (NMD ME) and mechanical insufflation-exsufflation (NMD MIE) techniques, mucus clearance exhibits a significant improvement under mucus viscosity reduction. In particular, the cough efficiency of NMD subjects with mechanical insufflation-exsufflation (NMD MIE) increases by approximately 20% when the mucus viscosity is one-quarter of its initial value.

## Conclusion

In this paper, the EWF model was applied to simulate the coughing clearance process in airways from generation 0 to generation 2 based on realistic geometry. Cough efficiency (CE) was adopted to evaluate the effect of a cough with or without assisted coughing techniques. The CE results for different mucus film thicknesses and viscosities were analyzed. A comparison of CE values among healthy, COPD and NMD models was conducted.

From the simulation results, we can see that:

1. In a healthy model, the mucus could be cleared efficiently through cough behavior without assisted coughing techniques. However, the COPD and NMD models would have difficulty in mucus clearance through cough without assisted coughing techniques.
2. The CE values increase with increasing mucus film thickness and decreasing mucus viscosity, which are consistent with empirical knowledge.
3. Assisted coughing techniques have little influence on the mucus clearance of COPD models. However, the mucus clearance of NMD models is greatly influenced by assisted coughing techniques. In particular, the CE of MIE is more than 40 times the value of unassisted coughing, which indicates that the MIE technique has a great effect on airway mucus clearance.

Although the cough process and CE could be predicted by the EWF model to some extent, the results should still be validated for a large amount of vivo data from clinics. The proposed model could be used to analyze a large number of clinical cases.

The deformation of airways during cough and viscoelastic properties of mucus were not considered in this study and will be our main research focus in the future.

## Supplementary information

Supplementary information

## Data Availability

The datasets generated and analysed during the current study are available in the Baidu cloud disk repository, https://pan.baidu.com/s/1zn-dc1p8Aqza_NcLcr-0jA. code: vrww.
