# Supplementary material for: Numerical Analysis of Airway Mucus Clearance Effectiveness Using Assisted Coughing Techniques
Source: Sci Rep. 2020 Feb 6;10:2030. doi: 10.1038/s41598-020-58922-7 (PMC7005022; doi:10.1038/s41598-020-58922-7)
Supplement: Supplementary file 1 — Supplementary information [file 41598_2020_58922_MOESM1_ESM.pdf]

SPRINGER NATURE

# Author Services

# Editing Certificate

This document certifies that the manuscript

## Numerical Analysis of Airway Mucus Clearance Effectiveness Using Assisted Coughing Techniques

prepared by the authors

Shuai Ren, Limin Hao, Lin Wang, Zihao Luo, Yan Shi, Jinglong Niu, Maolin Cai, Weiqing Xu, Zujin Luo

was edited for proper English language, grammar, punctuation, spelling, and overall style by one or more of the highly qualified native English speaking editors at SNAS.

This certificate was issued on **December 11, 2019** and may be verified on the SNAS website using the verification code **8E73-3D7D-DBAD-365D-A2A9**.

Neither the research content nor the authors' intentions were altered in any way during the editing process. Documents receiving this certification should be English-ready for publication; however, the author has the ability to accept or reject our suggestions and changes. To verify the final SNAS edited version, please visit our verification page at secure.authorservices.springernature.com/certificate/verify. If you have any questions or concerns about this edited document, please contact SNAS at support@as.springernature.com.

SNAS provides a range of editing, translation, and manuscript services for researchers and publishers around the world. For more information about our company, services, and partner discounts, please visit authorservices.springernature.com.
